# Safe Reading

**Published:** 1858-02

**Authors:** 


### SAFE READING.

"HAD I read *Sterne* more, and *Voltaire* less, I should have known that the world was wide enough for HAMILTON and me," were the pregnant words of AARON BURR, when he laid upon his bed of death. A man's books mould his character, and determine his destiny. Our earliest reading makes or mars us. And yet the mass of people allow their children to read any thing that comes along—newspaper, magazine, novel—and do not seem to have the most remote idea of exercising any control in that direction. Every parent who takes the trouble to think a moment must know, that there is more or less of moral, social, or religious poison in three-fourths of our current literature. We heard a man who wields a most prolific and a most admired and sententious pen, in a lecture to a city audience, unblushingly speak of "the ravings of the Koran, and the screams of the prophets:" thus, as we interpreted it, placing the Koran and the Bible on the same platform. And it is not a very uncommon thing to hear a peripatetic lecturer run a tilt at Christianity, its ministers, or its members, for the reward of the approbatory titter of a mob—all showing, that safe hearing, as well as safe reading, should be an object of parental search.

We do not know when our approbation went out with such unrestrained cordiality, as when reading an editorial in the *New York Observer*, of some weeks ago: "It is a sad truth, that truth's friends have so much confidence in truth's vitality and activity, that they trust to its own locomotive powers to get ahead, while error pushes its agencies with sleepless and untiring zeal."

It is as true in medicine as it is in morals; the knavish and the ignorant become fanatical, and propagate their phrenzies with an energy worthy of a better cause. They summon both the rostrum and the press to aid them in their purposes; and anon, volunteers, but a single remove from them in intelligence, crowd around, to repeat their errors, retail their poisons, or disseminate their inane productions.

A clerical correspondent, by whose labors in both hemispheres humanity has been made the better and the happier, writes under a late date:

"I am reminded, by the perusal, a day or two since, of the December number of the *Journal of Health*, that you are a very prompt and decided man. Promptness and decision are qualities which I like, and therefore, (it will not harm you, if I say it), while I like your Journal for its own sake, I like it because I like its editor. I regularly read every article in every number, and, though I may sometimes hesitate, or doubt, or even dissent, about some position pertaining to this or that subject, the vigor and raciness, and Saxon plainness of the language, together with its evident honesty and importance, make each number a blessing to me. It comes like a duly flavored and effervescing glass of soda, which, though like all such beverages, it calls for speedy attention, and necessitates an immediate disposal of it, nevertheless, unlike all others, it maintains its sparkle and its goodness marvellously.

"I wish you could administer some moral medicine to your brother editors, which might make their journals equally exhilarating, and, at the same time, equally safe and useful. Do you know, my dear sir, of any "Grand Specific," which will give "*health*" to our self-styled "*Religious* Newspapers," and make them what they profess to be? If there is anything that will make them "*Religious*," and perfectly safe, as the religious reading-matter of a family of children, not a few of us poor, simple, old-fashioned, country ministers would "rejoice with great joy exceedingly." When our secular papers, almost without exception, more or less openly insult or misrepresent evangelical religion, it seems too bad that our "Religious" papers should so sadly fail to breathe its spirit and exemplify its plainest principles. Many a Christian parent do I know who is almost afraid to have his family read any paper, lest the powerful agency of the press should infuse either poisoned teachings or a poisoned spirit into young, susceptible minds, ere he is aware of it.

"But I did not intend to write a lecture upon the Press; and my only apology for writing so much is, that my heart is surcharged, and, *currente calamo*, my fingers' ends would let out what was ready to overflow. I wanted, also, in all your endeavors to administer suitable medicine for *the mind and soul*, as well as for their important habitation, to bid you a hearty God-speed."

We propose, then, for the consideration of mature minds, that a new style of weekly reading be inaugurated, that newspapers should be printed of a religious character, which shall contain all the important recent news of the week in addition, with useful and honest advertisements, and let what is called the secular weekly be excluded from religious households, from every house where there is a family of children growing up; but it is to be understood, that every such religio-secular weekly be edited by a well-bred gentleman, as well as a Christian. This is certainly an important proviso; for as the editors of the religious press are almost universally clergymen, it is important that our children should regard them, through their editorial labor at least, as persons who conduct themselves "*with all lowliness and meekness, with long-suffering, forbearing one another in love*," and who, when they are "reviled, revile not again."

If there is a single editor of a religious newspaper, in these whole United States, who can humbly lay his hand on his heart, and say, "I am the man who fills up that description," we most sincerely envy him his place in the bosom of the Father.

We are far from saying, that there are not weekly newspapers which might not be read to advantage, and without injury, by a rising family; there are some in this city, there are many in the country, but for the sake of a broad line of demarkation, and to be wholly on the safe side, we have written as we have. If our suggestion is not practicable, then let no newspaper be handed to a child, which a conscientious parent has not first read and approved.

